# Supplementary material for: Assessment of the psychometric properties of self-management measurement instruments for individuals with type 2 diabetes: A systematic review protocol
Source: PLoS One. 2025 Aug 21;20(8):e0330448. doi: 10.1371/journal.pone.0330448 (PMC12370044; doi:10.1371/journal.pone.0330448)
Supplement: S1 File — (DOCX) [file pone.0330448.s001.docx]

**S1 SEARCH STRATEGY**

**Search strategy for MEDLINE (Ovid)**

| *n* | Search terms |
| --- | --- |
| #1 | exp Self-Management/ or Self-Management.mp. or (exp Self Care/ or Self Care.mp.) or (exp Self Efficacy/ or Self Efficacy.mp.) |
| #2 | exp Diabetes Mellitus/ or Diabetes Mellitus.mp. or (exp Diabetes Mellitus, Type 2/ or Diabetes Mellitus, Type 2.mp.) or Insulin Resistance/ or (diabetes mellitus, type ii or diabetes, type 2 or niddm).mp. |
| #3 | (instrument* or instruments* or measure* or measures* or questionnaire*).mp. or exp "Surveys and Questionnaires"/ or questionnaires*.mp. or scale*.mp. or scales*.mp. or tool*.mp. or tools*.mp. or survey*.mp. or test*.mp. |
| #4 | (instrumentation or methods).sh. OR (validation study or comparative study).pt. OR exp Psychometrics/ OR psychometr*.tw. OR (clinimetr* or clinometr*).mp. OR exp Outcome Assessment, Health Care/ OR outcome assessment.tw. OR outcome measure*.mp. OR exp Observer Variation/ OR observer variation.tw. OR exp Health Status Indicators/ OR exp Reproducibility of Results/ OR reproducib*.tw. OR exp Discriminant Analysis/ OR (reliab* or unreliab* or valid* or coefficient of variation or coefficient or homogeneity or homogeneous or internal consistency).tw. OR (cronbach* and (alpha or alphas)).tw. OR (item and (correlation* or selection* or reduction*)).tw. OR agreement.mp. OR precision.mp. OR imprecision.mp. OR precise values.mp. OR test-retest.tw. OR (test and retest).tw. OR (reliab* and (test or retest)).tw. OR stability.tw. OR (interrater or inter-rater or intrarater or intra-rater).tw. OR (intertester or inter-tester or intratester or intra-tester).tw. OR (interobserver or inter-observer or intraobserver or intra-observer).tw. OR (intertechnician or inter-technician or intratechnician or intra-technician).tw. OR (interexaminer or inter-examiner or intraexaminer or intra-examiner).tw. OR (interassay or inter-assay or intraassay or intra-assay).tw. OR (interindividual or inter-individual or intraindividual or intra-individual).tw. OR (interparticipant or inter-participant or intraparticipant or intra-participant).tw. OR kappa.tw. OR kappas.tw. OR repeatab*.mp. OR ((replicab* or repeated) and (measure or measures or findings or result or results or test or tests)).mp. OR (generaliza* or generalisa*).tw. OR concordance.tw. OR (intraclass and correlation*).tw. OR discriminative.tw. OR known group.tw. OR (factor analysis or factor analyses or factor structure or factor structures).tw. OR dimension*.tw. OR subscale*.tw. OR (multitrait and scaling and (analysis or analyses)).tw. OR item discriminant.tw. OR interscale correlation*.tw. OR (error or errors).tw. OR individual variability.tw. OR interval variability.tw. OR rate variability.tw. OR (variability and (analysis or values)).tw. OR (uncertainty and (measurement or measuring)).tw. OR standard error of measurement.tw. OR sensitiv*.tw. OR responsive*.tw. OR (limit and detection).tw. OR minimal detectable concentration.tw. OR interpretab*.tw. OR ((minimal or minimally or clinical or clinically) and (important or significant or detectable) and (change or difference)).tw. OR (small* and (real or detectable) and (change or difference)).tw. OR meaningful change.tw. OR ceiling effect.tw. OR floor effect.tw. OR item response model.tw. OR IRT.tw. OR rasch.tw. OR differential item functioning.tw. OR DIF.tw. OR computer adaptive testing.tw. OR item bank.tw. OR cross-cultural equivalence.tw. |
| #5 | #1 AND #2 AND #3 AND #4 |

**Search strategy for Web of Science**

| *n* | Search terms |
| --- | --- |
| #1 | (TS=(Self-Management) OR TS=(Self Management) OR TS=(Self-Care) OR TS=(Self Care) OR TS=(Self-Efficacy) OR TS=(Self Efficacy)) |
| #2 | (TS=(Diabetes Mellitus) OR TS=(non insulin depend) OR TS=(type 2 diabetes) OR TS=(type II diabetes) OR TS=(t2dm) OR TS=(niddm) OR TS=(glucose intolerance) OR TS=(insulin resistant) OR TS=(insulin resistance)) |
| #3 | (TS=(Instrument*) OR TS=(instruments*) OR TS=(measure*) OR TS=(measures*) OR TS=(questionnaire*) OR TS=(questionnaires*) OR TS=(scale*) OR TS=(scales*) OR TS=(tool*) OR TS=(tools*) OR TS=(survey*) OR TS=(test*)) |
| #4 | (TS=(instrumentation) OR TS=(methods) OR TS=(“validation stud*”) OR TS=(“comparative stud*”) OR TS=(psychometrics) OR TS=(psychometr*) OR ALL=(clinimetr*) OR ALL=(clinometr*) OR TS=(“outcome assessment”) OR TS=(“outcome measure”) OR TS=(“observer variation”) OR TS=(“observer variation”) OR TS=(“health status indicators”) OR TS=(“reproducib*”) OR TS=(“discriminant analysis”) OR TS=(reliab*) OR TS=(unreliab*) OR TS=(valid*) OR TS=(“coefficient of variation”) OR TS=(coefficient) OR TS=(homogeneity) OR TS=(homogeneous) OR TS=(“internal consistency”) OR ((TS=(alpha) OR TS=(alphas)) AND TS=(cronbach*)) OR ((TS=(correlation*) OR TS=(selection*) OR TS=(reduction*)) AND TS=(item)) OR TS=(agreement) OR TS=(precision) OR TS=(imprecision) OR TS=(precise values) OR TS=(test-retest) OR (TS=(test) AND TS=(retest)) OR ((TS=(test) OR TS=(retest)) AND TS=(reliab*)) OR TS=(stability) OR TS=(interrater) OR TS=(inter-rater) OR TS=(intrarater) OR TS=(intra-rater) OR TS=(intertester) OR TS=(inter-tester) OR TS=(intratester) OR TS=(intra-tester) OR TS=(interobserver) OR TS=(inter-observer) OR TS=(intraobserver) OR TS=(intra-observer) OR TS=(intertechnician) OR TS=(inter-technician) OR TS=(intratechnician) OR TS=(intra-technician) OR TS=(interexaminer) OR TS=(inter-examiner) OR TS=(intraexaminer) OR TS=(intra-examiner) OR TS=(interassay) OR TS=(inter-assay) OR TS=(intraassay) OR TS=(intra-assay) OR TS=(interindividual) OR TS=(inter-individual) OR TS=(intraindividual) OR TS=(intra-individual) OR TS=(interparticipant) OR TS=(inter-participant) OR TS=(intraparticipant) OR TS=(intra-participant) OR TS=(kappa) OR TS=(kappa’s) OR TS=(kappas) OR TS=(repeatab*) OR ((ALL=(replicab*) OR ALL=(repeated)) AND (ALL=(measure) OR ALL=(measures) OR ALL=(findings) OR ALL=(result) OR ALL=(results) OR ALL=(test) OR ALL=(tests))) OR TS=(generaliza*) OR TS=(generalisa*) OR TS=(concordance) OR (TS=(intraclass) AND TS=(correlation*)) OR TS=(discriminative) OR TS=(known group) OR TS=(“factor analysis”) OR TS=(“factor analyses”) OR TS=(“factor structure”) OR TS=(“factor structures”) OR TS=(dimension*) OR TS=(subscale*) OR ((TS=(analysis) OR TS=(analyses)) AND TS=(scaling) AND TS=(multitrait)) OR TS=(“item discriminant”) OR TS=(“interscale correlation*”) OR TS=(error) OR TS=(errors) OR TS=(“individual variability”) OR TS=(“interval variability”) OR TS=(“rate variability”) OR ((TS=(values) OR TS=(analysis)) AND TS=(variability)) OR ((TS=(measurement) OR TS=(measuring)) AND TS=(uncertainty)) OR TS=(“standard error of measurement”) OR TS=(sensitiv*) OR TS=(responsive*) OR (TS=(limit) AND TS=(detection)) OR TS=(“minimal detectable concentration”) OR TS=(interpretab*) OR ((TS=(minimal) OR TS=(minimally) OR TS=(clinical) OR TS=(clinically)) AND (TS=(important) OR TS=(significant) OR TS=(detectable)) AND (TS=(change) OR TS=(difference))) OR (TS=(small) AND (TS=(real) OR TS=(detectable)) AND (TS=(change) OR TS=(difference))) OR TS=(“meaningful change”) OR TS=(“ceiling effect”) OR TS=(“floor effect”) OR TS=(“Item response model”) OR TS=(IRT) OR TS=(Rasch) OR TS=(“differential item functioning”) OR TS=(DIF) OR TS=(“computer adaptive testing”) OR TS=(“item bank”) OR TS=(“cross-cultural equivalence”) OR TS=(“development”)) |
| #5 | #1 AND #2 AND #3 AND #4 |
| #6 | TS=(delphi-technique) OR TS=(cross-sectional) OR TS=(addresses) OR TS=(biography) OR TS=(case reports) OR TS=(comment) OR TS=(directory) OR TS=(editorial) OR TS=(festschrift) OR TS=(interview) OR TS=(lectures) OR TS=(legal cases) OR TS=(legislation) OR TS=(letter) OR TS=(news) OR TS=(newspaper article) OR TS=(patient education handout) OR TS=(popular works) OR TS=(congresses) OR TS=(consensus development conference) OR TS=(consensus development conference, nih) OR TS=((practice guideline)) OR TS=((animals NOT humans)) |
| #7 | #5 NOT #6 |

**Search strategy for Scopus**

| *n* | Search terms |
| --- | --- |
| #1 | (TITLE-ABS-KEY (“Self-Management” OR “Self Management” OR “Self-Care” OR “Self Care” OR “Self-Efficacy” OR “Self Efficacy”)) |
| #2 | (TITLE-ABS-KEY (“diabetes mellitus” OR “non insulin depend” OR “type 2 diabetes” OR “type II diabetes” OR “t2sm” OR “niddm” OR “glucose intolerance” OR “insulin resistant” OR “insulin resistance”)) |
| #3 | (TITLE-ABS-KEY (“Instrument*” OR “Instruments*” OR “measure*” OR “measures*” OR “questionnaire*” OR “questionnaires” OR “scale*” OR “scales*” OR “tool*” OR “tools*” OR  “survey*” OR “test*”)) |
| #4 | (TITLE-ABS-KEY ("instrumentation” OR “methods” OR “Validation Studies” OR “Comparative Study” OR “psychometrics” OR “psychometr*” OR “clinimetr*” OR “clinometr*” OR “outcome assessment (health care)” OR “outcome assessment” OR “outcome measure*” OR “observer variation” OR “observer variation” OR “Health Status Indicators” OR “reproducibility of results” OR “reproducib*” OR “discriminant analysis” OR “reliab*” OR “unreliab*” OR “valid*” OR “coefficient of variation” OR “coefficient” OR “homogeneity” OR “homogeneous” OR “internal consistency” OR (“cronbach*” AND “alpha” OR “alphas”) OR (“item” AND (“correlation*” OR “selection*” OR “reduction*”)) OR “agreement” OR “precision” OR “imprecision” OR “precise values” OR “test-retest” OR (“test” AND “retest”) OR (“reliab*” AND (“test” OR “retest”)) OR “stability” OR “interrater” OR “inter-rater” OR “intrarater” OR “intra-rater” OR “intertester” OR “inter-tester” OR “intratester” OR “intra-tester” OR “interobserver” OR “inter-observer” OR “intraobserver” OR “intra-observer” OR “intertechnician” OR “inter-technician” OR “intratechnician” OR “intra-technician” OR “interexaminer” OR “inter-examiner” OR “intraexaminer” OR “intra-examiner” OR “interassay” OR “inter-assay” OR “intraassay” OR “intra-assay” OR “interindividual” OR “inter-individual” OR “intraindividual” OR “intra-individual” OR “interparticipant” OR “inter-participant” OR “intraparticipant” OR “intra-participant” OR “kappa” OR “kappas” OR “repeatab*” OR ((“replicab*” OR “repeated”) AND (“measure” OR “measures” OR “findings” OR “result” OR “results” OR “test” OR “tests”)) OR “generaliza*” OR “generalisa*” OR “concordance” OR (“intraclass” AND “correlation*”) OR “discriminative” OR “known group” OR “factor analysis” OR “factor analyses” OR “factor structure” OR “factor structures” OR “dimension*” OR “subscale*” OR (“multitrait” AND “scaling” AND (“analysis” OR “analyses”)) OR “item discriminant” OR “interscale correlation*” OR “error” OR “errors” OR “individual variability” OR “interval variability” OR “rate variability” OR (“variability” AND (“analysis” OR “values”)) OR (“uncertainty” AND (“measurement” OR “measuring”)) OR “standard error of measurement” OR “sensitiv*” OR “responsive*” OR (“limit” AND “detection”) OR “minimal detectable concentration” OR “interpretab*” OR ((“minimal” OR “minimally” OR “clinical” OR “clinically”) AND (“important” OR “significant” OR “detectable”) AND (“change” OR “difference”)) OR (“small*” AND (“real” OR “detectable”) AND (“change” OR “difference”)) OR “meaningful change” OR “ceiling effect” OR “floor effect” OR “Item response model” OR “IRT” OR “Rasch” OR “Differential item functioning” OR “DIF” OR “computer adaptive testing” OR “item bank” OR “cross-cultural equivalence” OR “development”)) |
| #5 | #1 AND #2 AND #3 AND #4 |

**Search strategy for PsycINFO (APA)**

| *n* | Search terms |
| --- | --- |
| #1 | (“Self-Management” OR “Self Management” OR “Self-Care” OR “Self Care” OR “Self-Efficacy” OR “Self Efficacy”) |
| #2 | (“diabetes mellitus” OR “non insulin depend” OR “type 2 diabetes” OR “type II diabetes” OR “t2sm” OR “niddm” OR “glucose intolerance” OR “insulin resistant” OR “insulin resistance”) |
| #3 | (“Instrument*” OR “Instruments*” OR “measure*” OR “measures*” OR “questionnaire*” OR “questionnaires” OR “scale*” OR “scales*” OR “tool*” OR “tools*” OR  “survey*” OR “test*”) |
| #4 | ("instrumentation” OR “methods” OR “Validation Studies” OR “Comparative Study” OR “psychometrics” OR “psychometr*” OR “clinimetr*” OR “clinometr*” OR “outcome assessment (health care)” OR “outcome assessment” OR “outcome measure*” OR “observer variation” OR “observer variation” OR “Health Status Indicators” OR “reproducibility of results” OR “reproducib*” OR “discriminant analysis” OR “reliab*” OR “unreliab*” OR “valid*” OR “coefficient of variation” OR “coefficient” OR “homogeneity” OR “homogeneous” OR “internal consistency” OR (“cronbach*” AND “alpha” OR “alphas”) OR (“item” AND (“correlation*” OR “selection*” OR “reduction*”)) OR “agreement” OR “precision” OR “imprecision” OR “precise values” OR “test-retest” OR (“test” AND “retest”) OR (“reliab*” AND (“test” OR “retest”)) OR “stability” OR “interrater” OR “inter-rater” OR “intrarater” OR “intra-rater” OR “intertester” OR “inter-tester” OR “intratester” OR “intra-tester” OR “interobserver” OR “inter-observer” OR “intraobserver” OR “intra-observer” OR “intertechnician” OR “inter-technician” OR “intratechnician” OR “intra-technician” OR “interexaminer” OR “inter-examiner” OR “intraexaminer” OR “intra-examiner” OR “interassay” OR “inter-assay” OR “intraassay” OR “intra-assay” OR “interindividual” OR “inter-individual” OR “intraindividual” OR “intra-individual” OR “interparticipant” OR “inter-participant” OR “intraparticipant” OR “intra-participant” OR “kappa” OR “kappas” OR “repeatab*” OR ((“replicab*” OR “repeated”) AND (“measure” OR “measures” OR “findings” OR “result” OR “results” OR “test” OR “tests”)) OR “generaliza*” OR “generalisa*” OR “concordance” OR (“intraclass” AND “correlation*”) OR “discriminative” OR “known group” OR “factor analysis” OR “factor analyses” OR “factor structure” OR “factor structures” OR “dimension*” OR “subscale*” OR (“multitrait” AND “scaling” AND (“analysis” OR “analyses”)) OR “item discriminant” OR “interscale correlation*” OR “error” OR “errors” OR “individual variability” OR “interval variability” OR “rate variability” OR (“variability” AND (“analysis” OR “values”)) OR (“uncertainty” AND (“measurement” OR “measuring”)) OR “standard error of measurement” OR “sensitiv*” OR “responsive*” OR (“limit” AND “detection”) OR “minimal detectable concentration” OR “interpretab*” OR ((“minimal” OR “minimally” OR “clinical” OR “clinically”) AND (“important” OR “significant” OR “detectable”) AND (“change” OR “difference”)) OR (“small*” AND (“real” OR “detectable”) AND (“change” OR “difference”)) OR “meaningful change” OR “ceiling effect” OR “floor effect” OR “Item response model” OR “IRT” OR “Rasch” OR “Differential item functioning” OR “DIF” OR “computer adaptive testing” OR “item bank” OR “cross-cultural equivalence” OR “development”) |
| #5 | #1 AND #2 AND #3 AND #4 |

**Search strategy for Embase and CINAHL (EBSCOhost)**

| *n* | Search terms |
| --- | --- |
| #1 | (“Self-Management” OR “Self Management” OR “Self-Care” OR “Self Care” OR “Self-Efficacy” OR “Self Efficacy”) |
| #2 | (“diabetes mellitus” OR “non insulin depend” OR “type 2 diabetes” OR “type II diabetes” OR “t2sm” OR “niddm” OR “glucose intolerance” OR “insulin resistant” OR “insulin resistance”) |
| #3 | (“Instrument*” OR “Instruments*” OR “measure*” OR “measures*” OR “questionnaire*” OR “questionnaires” OR “scale*” OR “scales*” OR “tool*” OR “tools*” OR  “survey*” OR “test*”) |
| #4 | ("instrumentation” OR “methods” OR “Validation Studies” OR “Comparative Study” OR “psychometrics” OR “psychometr*” OR “clinimetr*” OR “clinometr*” OR “outcome assessment (health care)” OR “outcome assessment” OR “outcome measure*” OR “observer variation” OR “observer variation” OR “Health Status Indicators” OR “reproducibility of results” OR “reproducib*” OR “discriminant analysis” OR “reliab*” OR “unreliab*” OR “valid*” OR “coefficient of variation” OR “coefficient” OR “homogeneity” OR “homogeneous” OR “internal consistency” OR (“cronbach*” AND “alpha” OR “alphas”) OR (“item” AND (“correlation*” OR “selection*” OR “reduction*”)) OR “agreement” OR “precision” OR “imprecision” OR “precise values” OR “test-retest” OR (“test” AND “retest”) OR (“reliab*” AND (“test” OR “retest”)) OR “stability” OR “interrater” OR “inter-rater” OR “intrarater” OR “intra-rater” OR “intertester” OR “inter-tester” OR “intratester” OR “intra-tester” OR “interobserver” OR “inter-observer” OR “intraobserver” OR “intra-observer” OR “intertechnician” OR “inter-technician” OR “intratechnician” OR “intra-technician” OR “interexaminer” OR “inter-examiner” OR “intraexaminer” OR “intra-examiner” OR “interassay” OR “inter-assay” OR “intraassay” OR “intra-assay” OR “interindividual” OR “inter-individual” OR “intraindividual” OR “intra-individual” OR “interparticipant” OR “inter-participant” OR “intraparticipant” OR “intra-participant” OR “kappa” OR “kappas” OR “repeatab*” OR ((“replicab*” OR “repeated”) AND (“measure” OR “measures” OR “findings” OR “result” OR “results” OR “test” OR “tests”)) OR “generaliza*” OR “generalisa*” OR “concordance” OR (“intraclass” AND “correlation*”) OR “discriminative” OR “known group” OR “factor analysis” OR “factor analyses” OR “factor structure” OR “factor structures” OR “dimension*” OR “subscale*” OR (“multitrait” AND “scaling” AND (“analysis” OR “analyses”)) OR “item discriminant” OR “interscale correlation*” OR “error” OR “errors” OR “individual variability” OR “interval variability” OR “rate variability” OR (“variability” AND (“analysis” OR “values”)) OR (“uncertainty” AND (“measurement” OR “measuring”)) OR “standard error of measurement” OR “sensitiv*” OR “responsive*” OR (“limit” AND “detection”) OR “minimal detectable concentration” OR “interpretab*” OR ((“minimal” OR “minimally” OR “clinical” OR “clinically”) AND (“important” OR “significant” OR “detectable”) AND (“change” OR “difference”)) OR (“small*” AND (“real” OR “detectable”) AND (“change” OR “difference”)) OR “meaningful change” OR “ceiling effect” OR “floor effect” OR “Item response model” OR “IRT” OR “Rasch” OR “Differential item functioning” OR “DIF” OR “computer adaptive testing” OR “item bank” OR “cross-cultural equivalence” OR “development”) |
| #5 | #1 AND #2 AND #3 AND #4 |
| #6 | (“delphi-technique” OR “cross-sectional” OR “addresses” OR “biography” OR “case reports” OR “comment” OR “directory” OR “editorial” OR “festschrift” OR “interview” OR “lectures” OR “legal cases” OR “legislation” OR “letter” OR “news” OR “newspaper article” OR “patient education handout” OR “popular works” OR “congresses” OR “consensus development conference” OR “consensus development conference, nih” OR “practice guideline”) OR (“animals” NOT “humans”) |
| #7 | #5 NOT #6 |
