# Supplementary material for: Assessment of the psychometric properties of self-management measurement instruments for individuals with type 2 diabetes: A systematic review protocol
Source: PLoS One. 2025 Aug 21;20(8):e0330448. doi: 10.1371/journal.pone.0330448 (PMC12370044; doi:10.1371/journal.pone.0330448)
Supplement: Attachment — Submitted filename: Reviewed Manuscript PONE-D-25-17107.docx [file pone.0330448.s003.docx]

Thank you for the opportunity to contribute as a reviewer for this manuscript. I believe this review is important and valuable for advancing the field of T2DM care.

***Abstract:***

- It is a good, concise introduction. I think it would be better to be more clearly specifying the importance of self-management in relation to T2DM. The current version makes me understand that self-management is essential for disease control in general e.g. other chronic diseases (not specific to T2DM).
- The last sentence mentioning the use of ICF as a guide for data analysis which I think it might better fit under the “Methods” rather than “Context and Objectives”.

***Introduction:***

- The introduction is concise and easy to understand. However, in line 78-84, could you explain more the rationale for the need of this study according to the limitation of previous study (limitations regarding the inclusion of instruments for individuals with T2DM) and show how your review addresses this gap in knowledge.

***Methods:***

- ***Eligibility criteria***: I am wondering why study designs such as cross-sectional studies and post-intervention analyses will be excluded, and what study design or research type will be included in this review (e.g. psychometric study)? I understand that this review is focussing on the study targeting the development and validation of instrument. In case of the paper regarding instrument development were reported in qualitative study, would it be included or excluded?
- ***Search strategy***:
  - Would the hand search of the reference lists (backward citation tracking) of all study mean those study met eligibility criteria, or all study retrieved from databases?
  - I am thinking that forward citation tracking could also be beneficial for this review.
  - Well-structured and thorough search terms (according to Supplementary File 1).

***Certainty of evidence and instrument recommendation:*** The instrument recommendation within this review is very important and it will benefit research and practice in the field of T2DM.

***Data synthesis:***

- You mentioned that you will use a narrative synthesis. My thinking is that it would be good to give citation to make it clearer that which (version of) narrative synthesis you will be using. I also think it could be helpful for reader if you can provide a very short list/steps of narrative synthesis you are planning to do.
- It is a good point that you mentioned ***“If a validation study of the same instrument for different populations exists, its measurement properties results will be considered as a single instrument”***. I noticed that one of your inclusion criteria is to include cultural adaptation study and that I would assume that you might include this kind of study which may have different language versions of instrument. It would be good to mention this as well.

***Discussion***: Clear justification to conduct this review and provided implication of the result of this review.
