# Supplementary material for: Assessment of the psychometric properties of self-management measurement instruments for individuals with type 2 diabetes: A systematic review protocol
Source: PLoS One. 2025 Aug 21;20(8):e0330448. doi: 10.1371/journal.pone.0330448 (PMC12370044; doi:10.1371/journal.pone.0330448)
Supplement: Attachment — Submitted filename: Point_by_point_PLOS_T2DM.docx [file pone.0330448.s005.docx]

***PONE-D-25-17107***

***Manuscript entitled: “Assessment of the Psychometric Properties of Self-Management Measurement Instruments for Individuals with Type 2 Diabetes: A Systematic Review Protocol”.***

***Dear editor and reviewers,***

Thank you very much for your suggestion on our manuscript. We carefully considered your comments and revised the paper based on those comments and recommendations. We hope that these revisions improve the quality of the paper. You will find detailed responses to the comments made by you as well as those of the reviewers below.

**Editor(s)' Comments**

1. Please ensure that your manuscript meets PLOS ONE's style requirements, including those for file naming.

*Authors’ response: we appreciate your suggestion and ensure that we have checked all style requirements to meet the journal rules.*

2. Thank you for stating in your Funding Statement: [This study was partly financed by the Coordenação de Aperfeiçoamento de Pessoal de Nível Superior – Brasil (CAPES). Fiance Code 001.]. Please provide an amended statement that declares *all* the funding or sources of support (whether external or internal to your organization) received during this study, as detailed online in our guide for authors at http://journals.plos.org/plosone/s/submit-now. Please also include the statement “There was no additional external funding received for this study.” in your updated Funding Statement. Please include your amended Funding Statement within your cover letter. We will change the online submission form on your behalf.

*Authors’ response: We appreciate your concern. When we declare that “This study was partly financed by the Coordenação de Aperfeiçoamento de Pessoal de Nível Superior – Brasil (CAPES). Finance Code 001” we mean that Alexandre Almeida, as a post-graduation student, receives a scholarship that covers his PhD fees. There is no additional funding for this study as well as CAPES has no interference with the publications. It is worth highlighting that the statement is in accordance with the institution rules for publication. In this way we have included the following statement in the revised cover letter.*

***“This study was partly financed by the Coordenação de Aperfeiçoamento de Pessoal de Nível Superior – Brasil (CAPES). Finance Code 001. This funding source had no role in the preparation or decision of the manuscript . There was no additional external funding received for this study.”***

3. When completing the data availability statement of the submission form, you indicated that you will make your data available on acceptance. We strongly recommend all authors decide on a data sharing plan before acceptance, as the process can be lengthy and hold up publication timelines. Please note that, though access restrictions are acceptable now, your entire data will need to be made freely accessible if your manuscript is accepted for publication. This policy applies to all data except where public deposition would breach compliance with the protocol approved by your research ethics board. If you are unable to adhere to our open data policy, please kindly revise your statement to explain your reasoning and we will seek the editor's input on an exemption. Please be assured that, once you have provided your new statement, the assessment of your exemption will not hold up the peer review process.

*Authors’ response: we appreciate your concern and agree that if we had any data to share at this moment it would be worth having an established plan to make them public. However, as a systematic review protocol we have no data available yet as the search did not start. As soon as we start data collection we will make a data share plan. In this way we would like to apologize if we have made any mistake by filling in this submission section. Moreover, we kindly ask the editor to consider our explanation and disregard the information filled during the submission process.*

**Reviewer #1**

Thank you for the opportunity to contribute as a reviewer for this manuscript. I believe this review is important and valuable for advancing the field of T2DM care.

***Abstract:***

**Comment 1:** It is a good, concise introduction. I think it would be better to be more clearly specifying the importance of self-management in relation to T2DM. The current version makes me understand that self-management is essential for disease control in general e.g. other chronic diseases (not specific to T2DM).

*Authors’ response: We appreciate your suggestion and have added some other information regarding self-management for the T2DM population specifically. Such inclusion may be seen on page 02, lines 34-36.*

**Comment 2:** The last sentence mentioning the use of ICF as a guide for data analysis which I think it might better fit under the “Methods” rather than “Context and Objectives”.

*Authors’ response: We appreciate your concern with this information. However, one of the secondary aims of the study is to analyze the extracted content of the items and link them to the International Classification of Functioning, Disability, and Health (ICF). In this way, it is mentioned at the end of the introduction session where we describe the study aims (page 02, lines 36-39). Moreover, it is also mentioned in the methods session ( page 02, lines 51-54).*

***Introduction:***

**Comment 3:** The introduction is concise and easy to understand. However, in line 78-84, could you explain more the rationale for the need of this study according to the limitation of previous study (limitations regarding the inclusion of instruments for individuals with T2DM) and show how your review addresses this gap in knowledge.

*Authors’ response: We appreciate your suggestion. To make it clear we have better explained the limitations of the previous studies. Moreover, the methodology shows how we plan to fill these gaps as we are including generic instruments that included T2DM participants and have no language restrictions. Changes may be seen on page 03, lines 79-86.*

***Methods:***

***Comment 4:*** *(Eligibility criteria*) I am wondering why study designs such as cross-sectional studies and post-intervention analyses will be excluded, and what study design or research type will be included in this review (e.g. psychometric study)? I understand that this review is focussing on the study targeting the development and validation of instrument. In case of the paper regarding instrument development were reported in qualitative study, would it be included or excluded?

*Authors’ response: We appreciate your concern with this information. However, the eligibility, inclusion and exclusion criteria were constructed following the COSMIN guidelines, which recommends the exclusion of studies that use instruments to compare outcomes (such as cohort studies, clinical trials, cross-sectional studies, post-intervention analyses, and case-control studies). Moreover, according to COSMIN* “Including them would require a more extensive and time-consuming search to find all studies on the mediation properties of any instrument in the population of interest, in addition to being unfeasible to read all studies, since some instruments used may not be reported in their abstracts.”*

**Mokkink LB, Elsman EBM, Terwee CB. COSMIN guideline for systematic reviews of patient-reported outcome measures version 2.0. Qual Life Res. 2024 Nov;33(11):2929-2939. doi: 10.1007/s11136-024-03761-6. Epub 2024 Aug 28. PMID: 39198348; PMCID: PMC11541334.*

***Comment 5:*** *(Search strategy*) Would the hand search of the reference lists (backward citation tracking) of all study mean those study met eligibility criteria, or all study retrieved from databases? I am thinking that forward citation tracking could also be beneficial for this review. Well-structured and thorough search terms (according to Supplementary File 1).

*Authors’ response: We appreciate your question. Hand search will be performed in reference lists of all primary studies, systematic reviews, and gray literature to include potential studies. Such information is shown in the search strategy session.*

***Comment 6:*** *(Certainty of evidence and instrument recommendation)* The instrument recommendation within this review is very important and it will benefit research and practice in the field of T2DM.

*Authors’ response: We appreciate your comment and we also believe that we may bring some benefit to the practice in T2DM field.*

**Comment 7:** (Data synthesis) You mentioned that you will use a narrative synthesis. My thinking is that it would be good to give citation to make it clearer that which (version of) narrative synthesis you will be using. I also think it could be helpful for reader if you can provide a very short list/steps of narrative synthesis you are planning to do.

*Authors’ response: We appreciate your concern and agree that a list of steps may help the reader to understand such information. In this way, we have added on page 6, lines 190 to 195 of the revised manuscript the following paragraph:*

***“Additionally, the narrative synthesis will be conducted according to the following steps: 1) grouping studies by construct and measurement properties assessed; 2) describing the methodological quality of each study based on the COSMIN Risk of Bias checklist; 3) summarizing the results for each measurement property, including consistency across studies; and 4) drawing overall conclusions for each measurement property per instrument, in accordance to the COSMIN* and GRADE recommendations.”***

*Besides the narrative synthesis the measurement properties will be rated as s sufficient (+), insufficient (-), inconsistent (±), or indeterminate (?). The final rating of a measurement property will correspond to its lowest rating. The complete information is written on page 6, lines 196 to 200.*

**Mokkink LB, Elsman EBM, Terwee CB. COSMIN guideline for systematic reviews of patient-reported outcome measures version 2.0. Qual Life Res. 2024 Nov;33(11):2929-2939. doi: 10.1007/s11136-024-03761-6. Epub 2024 Aug 28. PMID: 39198348; PMCID: PMC11541334.*

**Comment 8:** It is a good point that you mentioned “If a validation study of the same instrument for different populations exists, its measurement properties results will be considered as a single instrument”. I noticed that one of your inclusion criteria is to include cultural adaptation study and that I would assume that you might include this kind of study which may have different language versions of instrument. It would be good to mention this as well.

*Authors’ response: We appreciate your concern. Such information is explained in the data synthesis session as shown bellow and on page 06, lines 185-188.*

*"If a validation study of the same instrument for different populations exists, its measurement properties results will be considered as a single instrument, but specific characteristics of each version will be discussed"*

***Discussion:***

**Comment 9:** Clear justification to conduct this review and provided implication of the result of this review.

*Authors’ response: Thank you for your comment.*

**Reviewer #2**

The manuscript is well-organized for a systematic review. The design follows recognized guidelines (COSMIN, PRISMA-P, and GRADE), reflecting good methodological rigor. However, there are areas that need improvement.

**Abstract:**

**Comment 1:** The objective is valid but could be rephrased to be clearer and more concrete. It would be ideal to add a final sentence summarizing what is expected to learn or contribute with this review. Additionally, including main results and conclusions is recommended.

*Authors’ response: we appreciate your suggestion. The aim was rephrased as seen in page 02, lines 36-39 as well as a discussion topic was added to explain the contributions of the review (page 02, lines 54-56). On the other hand we were not able to add results and conclusions as this is a systematic review protocol study.*

**Introduction:**

**Comment 2:** The study’s justification is relevant, but it could be enriched with a brief overview of existing instruments for self-management in T2DM, as well as a critical reflection on previous reviews. This would help better position the manuscript’s added value.

*Authors’ response: Thank you for your observation. We have made the recommended adjustments, improving the justification in a reflective way on what is available in the literature and the gaps that still exist (page 03, lines 79-86).*

**Methods:**

**Comment 3:** (Search strategy) It would be useful to specify an estimated cutoff date for the bibliographic searches.

*Authors’ response: We appreciate your suggestion however, considering that there is no date restriction for the protocol we did not include a cutoff point for it. Moreover, the final review will bring this information on the results session.*

**Comment 4:** (Contact with authors) Though mentioned later, it would be worth clarifying whether any contact was made with the authors of the reviewed articles and under what terms.

*Authors’ response: We appreciate your suggestion. However, considering that this is the systematic review protocol we did not start the search strategy. In this way we do not know yet if we will need to contact any authors or if all documents will be available. In case we need to contact any authors we will send an email asking for the full document.*

**Results:**

**Comment 5:** As a systematic review, it would be interesting to include the results of each search conducted across different databases, along with a brief outline of how the instruments will be classified.

*Authors’ response: Thank you for your concern. This information cannot be included as we have not started the searches yet. Since this is only a review protocol, we are following the schedule made during its registration at PROSPERO (CRD42024605840). However, the systematic review, when published, will bring all information regarding your questioning.*

**Discussion:**

**Comment 6:** The current section is somewhat generic. To strengthen it, we could delve deeper into the clinical and methodological implications of the study, anticipate potential limitations, and highlight how this review surpasses or complements previous work. Also, consider adding conclusions from the systematic review.

*Authors’ response: We appreciate your suggestion. The discussion section was rewritten according to suggestions and included more detailed information regarding the benefits of the review and its limitations. Such changes may be seen on page 7, lines 232-233 and page 8, lines 234-259. Regarding the suggestion to add conclusions from the systematic review we highlight that this is the review protocol. In this way, we have not started data collection and analysis which does not allow us to make any conclusion. Moreover, this study is a systematic review protocol and similar studies already published present the topic of discussion superficially, since there is no data to be discussed and even no conclusion.*

**Comment 7:** Finally, the basis for classifying the reviewed instruments and their analysis is unclear.

*Authors’ response: We appreciate your concern. We highlight that each instrument will be classified and justified into one of three categories according to the COSMIN recommendations as well as their analysis. Based on this recommendation the categories are grouped as: (A) The instrument is recommended for use, and the results are reliable; (B) The instrument may be recommended but requires further research to assess its quality; (C) The instrument should not be recommended. We will also provide suggestions based on feasibility considerations. This information is shown on page 7, lines 219-224. The reference used to classify the categories is shown bellow:*

*Mokkink LB, Elsman EBM, Terwee CB. COSMIN guideline for systematic reviews of patient-reported outcome measures version 2.0. Qual Life Res. 2024 Nov;33(11):2929-2939. doi: 10.1007/s11136-024-03761-6. Epub 2024 Aug 28. PMID: 39198348; PMCID: PMC11541334.*

**Reviewer #3**

**General assessment:**

**Comment 1:** This is a well-designed and timely protocol for a systematic review of self-management instruments in type 2 diabetes mellitus (T2DM), including an evaluation of their psychometric properties and linkage to the International Classification of Functioning, Disability and Health (ICF). The protocol is clearly structured, adheres to COSMIN guidelines, and proposes a comprehensive search strategy.

*Authors’ response: Thank you for your comment.*

**Comment 2:** The inclusion of instrument databases such as PROMIS and PROQOLID is a notable strength, and the authors’ decision to assess content via ICF coding adds an innovative dimension. The protocol addresses an important topic with practical implications for clinical and research contexts, particularly in the identification of reliable and valid tools for assessing self-management in chronic illness.

*Authors’ response: Thank you for your comment.*

**Major points for clarification and improvement:**

**Comment 3:** (Instruments with subscales for self-management) The exclusion of instruments measuring more than one construct is understandable; however, many validated instruments include distinct subscales for self-management. It is unclear whether such tools will be excluded altogether or considered based on the relevance of the subscale. The authors are encouraged to clarify this point, as it could impact the comprehensiveness of the review.

*Authors’ response: We appreciate your concern. Considering the study aims the authors believe that instruments that only have self-management as a subscale should also be excluded due to the possibility of covering a very large number of instruments and including heterogeneous instruments, making our analysis difficult. We have added this information on page 4, lines 115 and 116.*

**Comment 4:** (Choice of databases – rationale for excluding Cochrane and PEDro) The protocol omits both the Cochrane Library and PEDro, despite their relevance. Cochrane CENTRAL could include validation studies nested within trials, and PEDro may contain relevant psychometric evaluations in physiotherapy contexts. If these databases were deliberately excluded, the authors should briefly justify this decision.

*Authors’ response: Thank you for your observation. We have decided not to use such databases as we will exclude all studies associated with trials and other types of studies mentioned in the text (page 4, lines 113-117) according to eligibility criteria, which were constructed following COSMIN recommendations. The guideline recommends excluding studies that use instruments as a way to assess outcomes. Moreover, these studies would require a much more extensive search strategy, since search filters for measurement properties cannot be used, thus, the approach is not considered viable. We highlight that such criteria are used by several review studies on measurement instruments.*

**Comment 5:** (Lack of explicit quality thresholds for instrument evaluation) While the authors refer to COSMIN and GRADE frameworks, there is no indication of the thresholds that will be used to assess whether an instrument demonstrates adequate reliability, validity, or responsiveness (e.g., Cronbach’s alpha ≥ 0.70). Including these definitions would strengthen the transparency and reproducibility of the review.

*Authors’ response: We appreciate your concern. Methodological quality will be assessed by using the *COSMIN Risk of Bias checklist, which includes 116 items. These items assess all measurement properties, including Cronbach's alpha ≥ 0.70. Moreover, this tool provides step-by-step instructions on how the quality should be evaluated which may facilitate the construction of the review and reduce the risk of bias in the study. Such information is shown on page 5, lines 167-169 and page 6, lines 170-175.*

**Mokkink LB, de Vet HCW, Prinsen CAC, Patrick DL, Alonso J, Bouter LM, Terwee CB. COSMIN Risk of Bias checklist for systematic reviews of Patient-Reported Outcome Measures. Qual Life Res. 2018 May;27(5):1171-1179. doi: 10.1007/s11136-017-1765-4. Epub 2017 Dec 19. PMID: 29260445; PMCID: PMC5891552.*

**Comment 6:** (Handling of shortened or alternate versions of instruments) The protocol does not specify how the authors will deal with instruments that have short forms or multiple validated versions. Will these be reviewed separately, combined, or will only the original versions be considered? This decision should be made explicit.

*Authors’ response: Thank you for your comment. In case of validation studies of the same instrument for different populations or languages, its measurement properties results will be considered as a single instrument, but the specific characteristics of each version will be discussed separately as recommended by COSMIN. Moreover, this methodology is similar to instruments’ reviews already published. This information can be checked on page 6, lines 185-188.*

**Comment 7:** (Purpose and operationalization of ICF linkage) Although the plan to link instrument content to the ICF is an interesting innovation, the rationale and utility of this approach remain vague. Will this linkage be used to recommend instruments for specific clinical needs or populations? How will it inform the interpretation or selection of tools?

*Authors’ response: We appreciate your concern and also believe that the ICF linkage is an innovation that’s why we have considered it as a secondary aim. Moreover, taking into account that the ICF is a tool that describes and classifies health-related functionality and disability and is standardized worldwide, we believe that by identifying the instruments with the greatest similarity to the ICF can be taken into consideration when choosing the best instrument. Besides that we would like to identify gaps in the language of the instruments present in the literature for clinical and research use. In this way, we believe that this review will bring a combination of good methodological review, assessed measurement properties as well as instruments. Then the reader will be able to choose the best instrument based on a methodological review, assessed measurement properties as well as an instrument with standard language for functionality according to the ICF.*

**Comment 8:** (Language strategy in search procedure) The authors state that there will be no language restrictions, but the search strategies do not indicate how non-English terms will be incorporated. Will search terms be translated into other languages? Will translators be used to assess studies in different languages? Clarifying the technical implementation of this inclusive strategy would be helpful.

*Authors’ response: We appreciate your concern. Considering most of the published manuscripts present at least the abstract and keywords in English we will incorporate only the English terms as previously mentioned, without any translation to other languages. If the search is positive to a manuscript in a language other than English a translator fluent in the language will be contacted.*

**Comment 9:** (Lack of data sharing plan) The protocol does not specify whether and where the extracted data, quality assessments, or ICF mappings will be made available upon completion of the review. Including a plan for data sharing (e.g. via OSF, Figshare, or journal supplements) would align the protocol with open science practices and enhance its utility for researchers and practitioners.

*Authors’ response: We appreciate your concern. We will follow the schedule proposed at the moment of the registration at PROSPERO which means the systematic review will be finished in July, 2026. However, we did not choose the journal yet. Moreover, data sharing will be done in accordance with the guidelines of the journal in which the final version is published. For example, PLOS ONE has a statement and requires a sharing plan in the final version. As for the quality assessments and ICF mappings, they will also be made available together with the extracted data.*

**Comment 10:** This is a promising and well-constructed protocol with strong methodological foundations. Addressing the six points listed above would enhance its clarity, transparency, and completeness, ensuring the resulting review is as rigorous and comprehensive as possible.

*Authors’ response: Thank you for your comment and your observations made in our study.*
